# Transcription Factor HOXA9 is Linked to the Calcification and Invasion of Papillary Thyroid Carcinoma

**DOI:** 10.1038/s41598-019-43207-5

**Published:** 2019-05-01

**Authors:** Yilan Jin, Hyeung Kyoo Kim, Jeonghun Lee, Euy Young Soh, Jang-Hee Kim, Insun Song, Yoon-Sok Chung, Yong Jun Choi

**Affiliations:** 1 Department of Endocrinology and Metabolism, Ajou University School of Medicine, Suwon, South Korea; 2 Department of Surgery, Ajou University School of Medicine, Suwon, South Korea; 3 Department of Pathology, Ajou University School of Medicine, Suwon, South Korea; 4 School of Biological Sciences, Seoul National University, Seoul, South Korea

**Keywords:** Thyroid cancer, Cell invasion

## Abstract

Calcification is important for the diagnosis of papillary thyroid carcinoma (PTC). Runt-related transcription factor 2 (RUNX2), a master transcription factor associated with osteogenic differentiation, is reportedly related to PTC calcification and invasiveness. However, its regulatory role in this process is somewhat uncharacterized. Here, we attempted to identify genes that regulate RUNX2 and clarify its function in PTC carcinogenesis and calcification. The expression of RUNX2-upstream genes was evaluated by real-time PCR in Nthy-Ori 3-1 normal thyroid cells and TPC1 and BHP10-3 PTC cell lines. Luciferase and chromatin immunoprecipitation assays were performed with candidate genes after cloning the *RUNX2* promoter. We found that *RUNX2* promoter activity was enhanced by homeobox family A9 (HOXA9). Over-expression of HOXA9 was found to enhance alkaline phosphatase activity, mineralization, and *in vitro* tumour cell migration and invasion, whereas downregulation had the opposite effects. These results indicate that HOXA9, a positive regulator of RUNX2, can enhance calcification, migration, and invasion in PTC. Our data improve the understanding of the molecular mechanisms of microcalcification in PTC as well as tumorigenesis.

## Introduction

Papillary thyroid carcinoma (PTC) is the most common type of malignant thyroid tumour^1–3^. Calcifications, characteristic of this disease, are important for the diagnosis of PTC^4,5^. However, these are frequently found not only in cancer tissues but also in some benign nodules^6^. Psammoma bodies (PBs), concentric lamellated calcified structures, are most common in histological sections of PTC^7,8^. PBs are largely considered the result of dystrophic calcification, in which deposition occurs locally in nonviable or dying tissues^9^. However, rather than being the outcome of dystrophic calcification of dead or dying tissue, it has been suggested that PBs might represent an active biologic process leading to the degeneration/death of tumour cells and the retardation of growth of the neoplasm^9^. Thus, the underlying mechanism and role of calcification in PTC are not fully understood.

Runt-related transcription factor 2 (RUNX2) belongs to the RUNX family and consists of three isoforms, namely types I, II, and III^10^. The main function of type I and II RUNX2 is as transcription factors involved in osteoblast differentiation and bone formation^11,12^. However, they are also involved in the carcinogenesis of breast and prostate cancers, as well as bone metastasis^13,14^. Moreover, some findings have suggested that high RUNX2 expression can predict breast cancer recurrence^15^ or induce myeloma progression in the bone^16^. RUNX2 also plays a critical role in mesenchymal cell differentiation to the osteogenic lineage^17^.

Recently, it has been reported that thyrocytes show similar characteristics to osteoblasts and that the expression of RUNX2 is increased in PTC tissues^18^. Further, PTC patients with microcalcifications were found to express significantly higher levels of *RUNX2* mRNA in serum than those without microcalcifications^17^. Enhanced RUNX2 signalling has been functionally linked to tumour invasion and metastasis in thyroid carcinoma by regulating epithelial-to-mesenchymal transition-related molecules, matrix metalloproteinases, and angiogenic/lymphangiogenic factors^19^. However, the regulatory role of RUNX2 in thyroid calcification and carcinogenesis has not been fully elucidated.

In this study, our aim was to discover a novel protein that regulates the expression of RUNX2 and to clarify the function of this marker and RUNX2 in carcinogenesis and calcification. For this, we screened several candidate transcription factors, upstream genes of RUNX2, and homeobox A9 (HOXA9) was identified as a potential candidate. Hox proteins, a group of homeodomain-containing transcription factors, play a key role in oncogenesis and are extremely dysregulated both in solid and haematological malignancies^20–22^. The expression of HOXA9, as a member of the HOX gene family, is usually altered in solid cancers^23^. Thus, we elucidated the relationship between HOXA9 and RUNX2 and the associated functions in PTC carcinogenesis and calcification.

## Results

### HOXA9 regulates *RUNX2* gene expression

**Figure 1:**
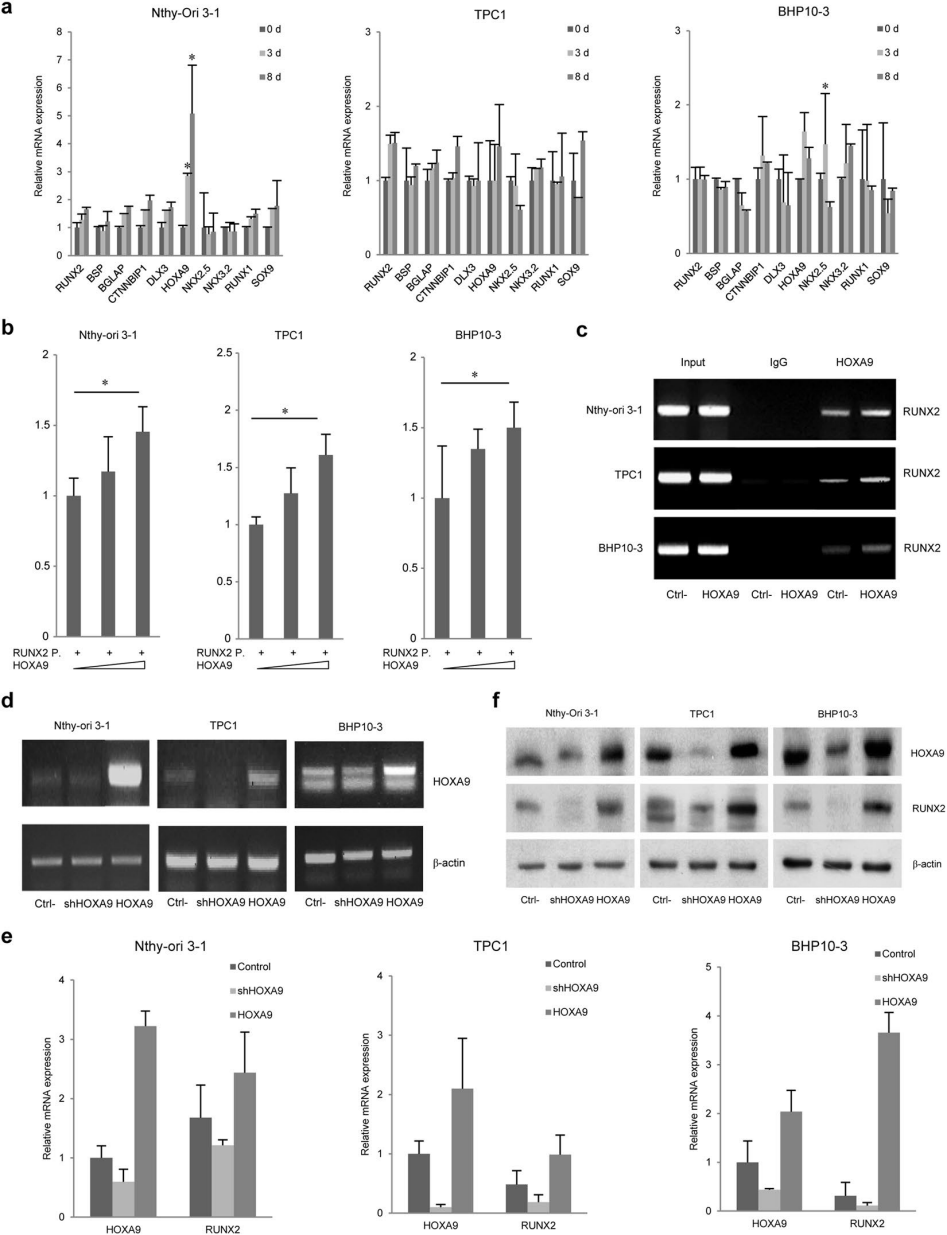
Expression of HOXA9 and RUNX2 in thyroid cells. (**a**) The expression of several selected candidate genes (RUNX2-upstream genes) in thyroid cell lines cultured in osteogenesis medium for 0, 3, and 8 days, as well as *HOXA9* expression, was evaluated by real-time PCR. (**b**,**c**) The *RUNX2* promoter (P) was cloned and plasmid DNA encoding HOXA9 was transfected into thyroid cells; HOXA9-binding activity and ability at the *RUNX2* promoter region was assessed by luciferase reporter assays and chromatin immunoprecipitation (ChIP) assays. (**d**) HOXA9 was knocked down or overexpressed in two types of thyroid cell lines. The RNA expression of *HOXA9* was assessed by qRT-PCR. (**e**,**f**) Alterations to the RNA and protein expression of RUNX2 depending on HOXA9 levels. Error bars represent standard deviation (n = 3 biological replicates). *p < 0.05. The grouping of blots cropped from different parts of the same gel. The full-length blots are included in a Supplementary Information file (Supplementary Fig. S1).

To discover a novel protein that regulates the expression of RUNX2, we selected seven candidates (catenin beta interacting protein 1 (CTNNBIP1), distal-less homeobox 3 (DLX3), HOXA9, NK2 homeobox 5 (NKX2-5), —> NK3 homeobox 2 (NKX3-2), runt related transcription factor 1 (RUNX1), and SRY-Box 9 (SOX9)) from a transcription factor (TF) search website (http://www.cbrc.jp/research/db/TFSEARCH.html). Then, we screened the effect of candidates on osteoblastic marker genes including *RUNX2*, integrin binding sialoprotein (*IBSP*), and bone gamma-carboxyglutamic acid-containing protein (*BGLAP*) by measuring mRNA expression levels by real-time PCR. Total RNA was prepared from the normal cell line Nthy-Ori 3-1 and PTC cell lines TPC1 and BHP10-3, which were cultured in osteogenesis differentiation media for 0, 3, or 8 days; RNA from these cells was used as a template for real-time PCR (Fig. 1a). Based these data, HOXA9, for which expression changed over time with RUNX2, was selected as a primary candidate.

Subsequently, we cloned the *RUNX2* P1 promoter and performed luciferase reporter assays to investigate the regulatory interaction between RUNX2 and HOXA9. The promoter activity of *RUNX2* P1 was increased with the addition of HOXA9 (Fig. 1b). Next, we performed chromatin immunoprecipitation (ChIP) assays to assess the binding of HOXA9 to the promoter of *RUNX2* using both normal Nthy-Ori 3-1 cells and TPC1 and BHP10-3 PTC lines, with an anti-HOXA9 antibody. Consistent with luciferase assay data, the binding of HOXA9 to the *RUNX2* promoter improved in a dose-dependent manner (Fig. 1c). We also confirmed that *RUNX2* gene expression was upregulated or downregulated depending on HOXA9 expression. These results suggest that HOXA9 regulates *RUNX2* by binding its promoter in two types of thyroid cell lines, specifically control and PTC (Figs 1d–f and S1).

### HOXA9 mediates the calcification of thyroid cells

**Figure 2:**
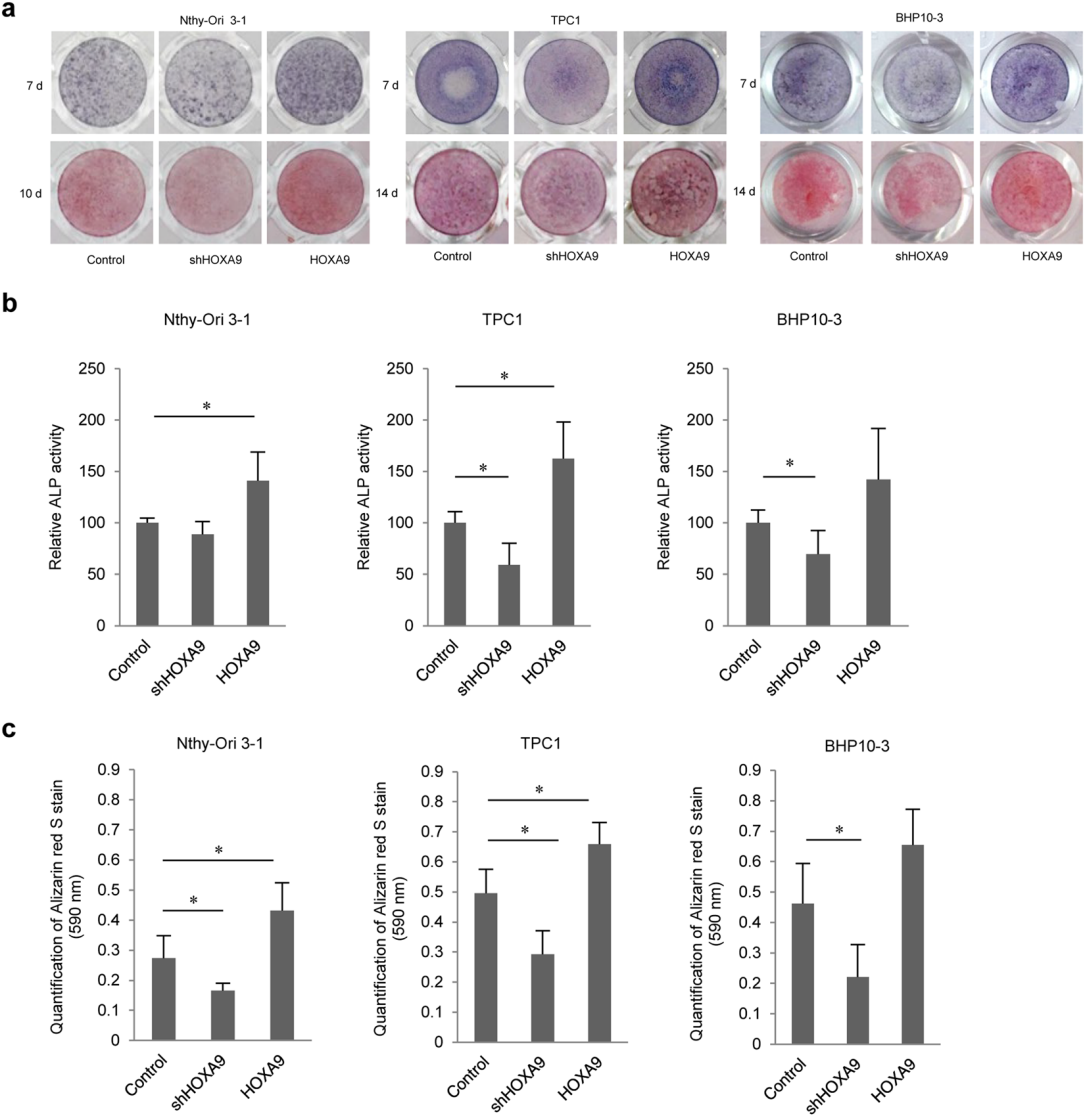
Effect of HOXA9 on osteoblast differentiation. (**a**) Alkaline phosphatase (ALP) staining (upper layer) was performed after 7 days and Alizarin red S staining was carried out on the 10^th^ day using Nthy-Ori 3-1 cells and on the 14^th^ day using papillary thyroid carcinoma (PTC) cells (TPC1 and BHP10-3). (**b**) ALP activity was measured at 405 nm using alkaline phosphatase yellow (pNPP) liquid substrate system with control, shHOXA9, and HOXA9-overexpressing cells for the two types of thyroid cells. (**c**) Alizarin red S-stained cells were extracted using cetylpyridinium chloride, and the mineralization level was quantified by measuring absorbance at 562 nm. Error bars represent standard deviation (n = 3 biological replicates). *p < 0.05.

To assess whether HOXA9 is involved in the process of calcification, alkaline phosphatase (ALP) staining was performed at 3, 5, and 7 days and Alizarin red S (ARS) staining was detected at 7–14 days. ALP activity was significantly enhanced in HOXA9-overexpressing Nthy-Ori 3-1 and TPC1 cells lines. In contrast, ALP activities were significantly reduced by the knockdown of this gene in TPC1 and BHP10-3 cell lines (Fig. 2a,b). Moreover, mineralization status was increased in HOXA9-overexpressing Nthy-Ori 3-1 and TPC1, but was attenuated in all HOXA9-knockdown groups (Fig. 2a,c). These data suggest that HOXA9 is involved in the process of thyroid calcification

### HOXA9 is associated with thyroid cell migration and invasion

**Figure 3:**
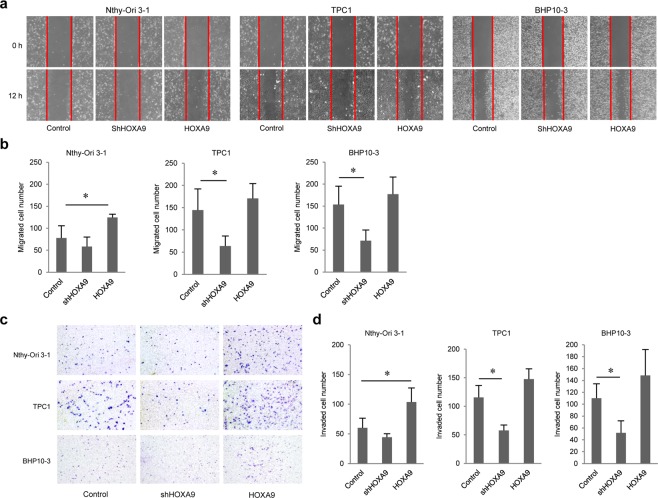
Migration and invasion ability of thyroid cells based on HOXA9 expression. (**a**) Thyroid cells were seeded in 6-well plates (6 × 10^4^ cells/well) with 2 ml of complete growth media. After 24 h, wound-healing assays were performed, and the wells were photographed every 12 h to monitor wound closure. (**c**) Thyroid cells were transferred to the top of Matrigel-coated chambers with 3 × 10^4^ cells/well in serum-free media. After 24 h of incubation, the invaded cells were fixed with methanol and stained with 0.5% crystal violet. (**b,d**) The numbers of migrating cells into the wound and invading cells were counted under a microscope. Experiments were repeated three times. Error bars represent standard deviation (n = 3 biological replicates). *p < 0.05.

To assess Nthy-Ori 3-1, TPC1, and BHP10-3 cell migration and invasion, wound healing and invasion assays were performed. HOXA9-overexpressing cells migrated more than control cells, whereas in the normal Nthy-Ori 3-1 cell line, HOXA9 knockdown suppressed migration; however, this difference was not significant. In PTC cells, wound healing ability was increased marginally in HOXA9-overexpressing cells compared to that in control cells and was decreased in HOXA9-knockdown cells (Fig. 3a,b). Furthermore, cell invasion was enhanced in HOXA9-overexpressing groups and reduced in HOXA9-knockdown groups (Fig. 3c,d). These data indicate that HOXA9 can mediate migration and invasion in two types of thyroid cells.

### HOXA9 enhances PTC calcification and tumour invasion directly or indirectly via RUNX2

**Figure 4:**
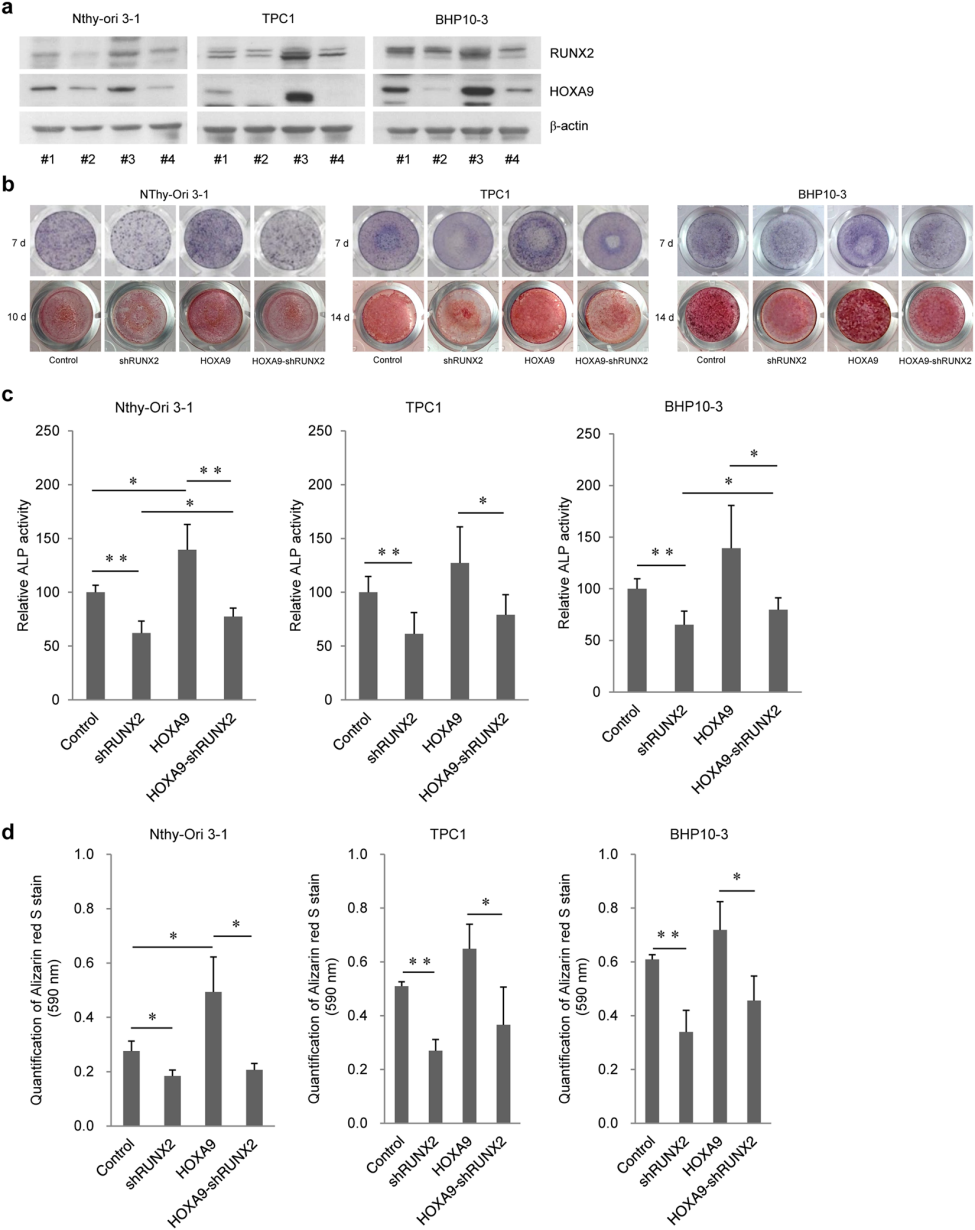
Effect of HOXA9 overexpression and shRUNX2 on osteoblast differentiation. (**a**) RUNX2 was knocked down in three control and HOXA9-overexpressing cell lines. Protein expression was assessed by western blotting (#1: Control, #2: shRUNX2, #3: HOXA9-overexpressing, #4: HOXA9-overexpressing/shRUNX2). The grouping of blots cropped from different parts of the same gel. The full-length blots are included in a Supplementary Information file (Supplementary Fig. S2). (**b**) Alkaline phosphatase (ALP) staining (upper layer) on the 7^th^ day and Alizarin red S staining (lower layer) on the 10^th^ day for Nthy-Ori 3-1 cells and on the 14^th^ day for papillary thyroid carcinoma (PTC) cells (TPC1 and BHP10-3). (**c**,**d**) Quantitative data for ALP and ARS staining in thyroid cells with control, shRUNX2, HOXA9-overexpressing, and HOXA9-overexpressing/shRUNX2 conditions. Error bars represent standard deviation (n = 3 biological replicates). *p < 0.05, **p < 0.005.

To determine whether the HOXA9-mediated effect on enhanced calcification and carcinogenesis is associated with RUNX2, we utilized a RUNX2-knockdown system and HOXA9-overexpression system with the normal cell line Nthy-Ori 3-1 and PTC cell lines TPC1 and BHP10-3 (Figs 4a and S2).

Next, we performed mineralization assays, as previously described, on these cells. ALP activities were significantly decreased in all RUNX2-knockdown cells (Fig. 4b,c). Further, mineralization was also suppressed in all RUNX2-downregulated groups (Fig. 4b,d). ALP activity in Nthy-Ori 3-1 and BHP10-3 cells was marginally enhanced with RUNX2 knockdown and HOXA9 overexpression compared to that in RUNX2-knockdown control cells, and this difference was statistically significant (*p* < 0.05 or *p* < 0.005; Fig. 4c).

**Figure 5:**
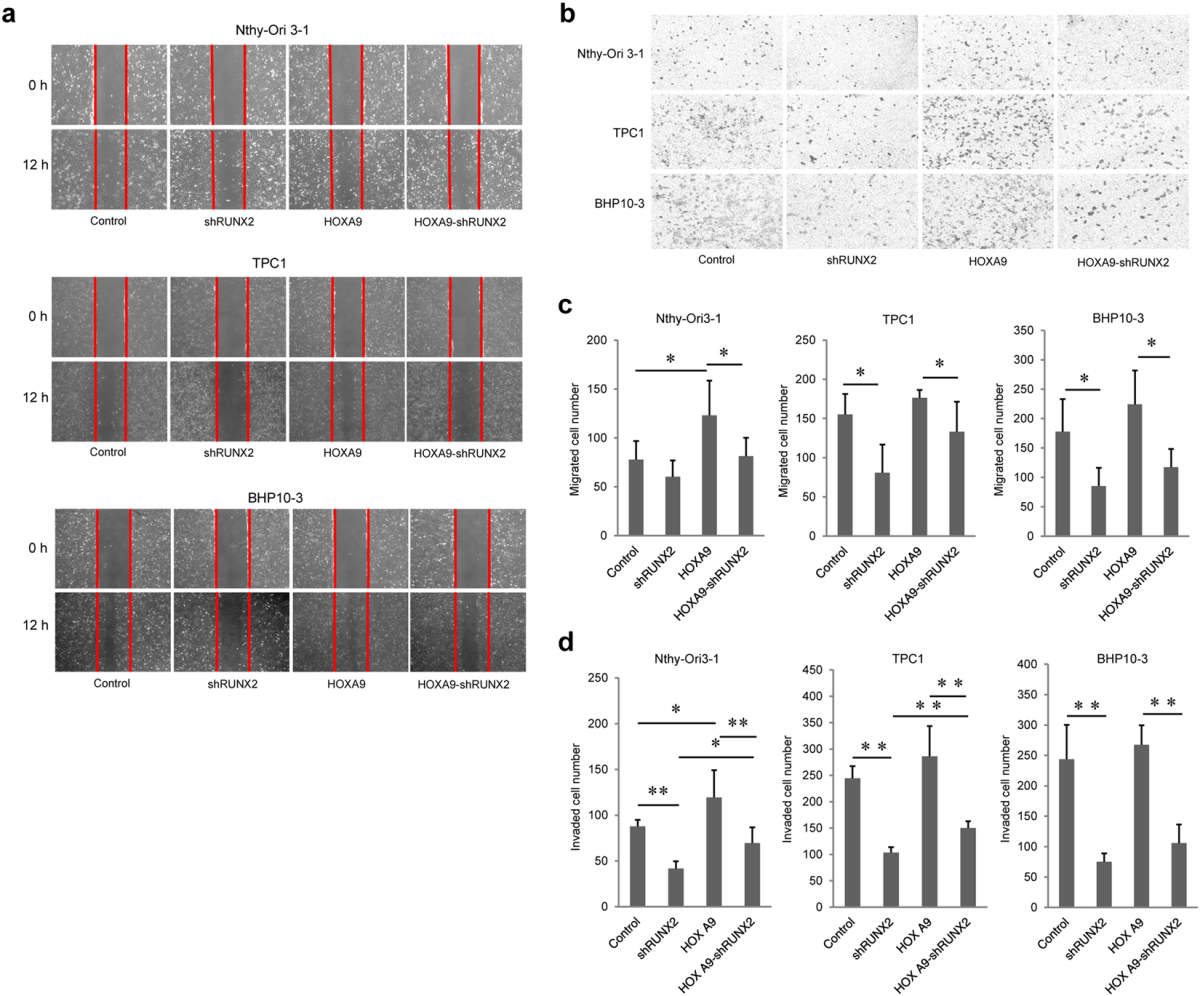
Migration and invasion ability based on HOXA9 and RUNX2 expression in thyroid cells. (**a**) Migration and invasion ability were demonstrated by wound-healing and transwell assays. Cells were seeded in 6-well plates (6 × 10^4^ cells/well), and after 24 h, wound-healing assays were performed and the wells were photographed every 12 h to monitor wound closure. (**b**) Thyroid cells were transferred to the top of Matrigel-coated chambers with 3 × 10^4^ cells/well in serum-free media. After 24 h of incubation, the invaded cells were fixed with methanol and stained with 0.5% crystal violet. (**c**) The number of cells that migrated into the wound. (**d**) The number of cells that invaded through the filter. The numbers of migrated cells into the wound and invaded cells were counted under a microscope. Experiments were repeated three times. Error bars represent standard deviation (n = 3 biological replicates). *p < 0.05, **p < 0.005.

Subsequently, wound healing and transwell assays were performed. Similar to the results of mineralization assays, migration was considerably decreased in RUNX2-knockdown cells compared to that in controls (Fig. 5a,c). Likewise, RUNX2 knockdown also suppressed invasion in the three thyroid cell lines. Additionally, invasion was enhanced in the groups of RUNX2-knockdown cells with HOXA9 overexpression compared to that in respective RUNX2-knockdown control cells (Fig. 5b,d).

These data suggest that HOXA9, as a positive regulator of RUNX2, can enhance calcification and tumour migration and invasion in PTC, dependent and independent of RUNX2.

## Discussion

In this study, we screened upstream mediators of RUNX2, and found one candidate, HOXA9, which regulates the expression of this gene in thyroid cell lines. Overexpression of HOXA9 was found to enhance ALP activity and mineralization, as well as *in vitro* tumour cell migration and invasion, whereas downregulation of this marker inhibited these processes. Moreover, cells exhibited enhanced migration and invasion in RUNX2-knockdown cells with HOXA9 overexpression compared to those in RUNX2-knockdown control cells. This suggests that HOXA9 could be linked to the calcification and tumour invasion of PTC, which is both independent and dependent of RUNX2.

RUNX2 is the master regulator in osteoblast differentiation^24–27^. It was also reported to have a role in thyroid carcinogenesis. Specifically, levels of this marker were found to be significantly higher in larger PTC tumors^25^. Moreover, RUNX2 regulates cellular invasion in thyroid tumour cells^26^. However, there have been few reports regarding regulators of RUNX2 during thyroid calcification and carcinogenesis. Accordingly, we identified one candidate, HOXA9, and confirmed that it regulates the expression of RUNX2 in thyroid cell lines. Homeobox (HOX) family genes encode a class of transcription factors that regulate the expression of numerous genes, control cell growth, and drive specific tissue differentiation^28^. Further, the expression of HOX family genes is often dysregulated in tumours^22^. HOXA9, as a transcription factor, is most commonly altered in solid tumours^23^. To demonstrate the function of HOXA9 in PTC calcification, we performed ALP and ARS staining assays. ALP activity and calcification were enhanced in HOXA9-overexpressing Nthy-Ori 3-1 and TPC1 cells and reduced in HOXA9-knockdown TPC1 and BHP10-3 cells. These results indicated that calcification could be regulated by HOXA9 in thyroid cell lines.

Many studies have established the function of HOXA9 in cancer cells. There are some reports indicating that HOXA9 knockdown can significantly decrease colony formation, invasion, and migration in colorectal cancer cells^29–31^. However, some others reported that HOXA9 inhibits migration and that the hypermethylation of *HOXA9* is especially apparent in the early stages of lung cancer^32–34^. Moreover, low expression of this marker was observed in cervical cancer cells and proliferation and migration were suppressed when HOXA9 expression was restored in these cells^35^. This protein was also found to restrict cell growth, survival, and invasion in T4-2 breast cancer cells^36^. In contrast, it was determined that HOXA9 promotes the viability and aggressiveness of glioblastoma cells^37^. Further, in ovarian cancer, this factor was shown to induce peritoneal macrophages to obtain an M2 tumour-promoting phenotype^38^. Likewise, HOXA9 encourages epithelial ovarian cancer growth in mouse xenograft models and supports the generation of a microenvironment for tumour growth^39^. However, there have been no reports regarding the role of HOXA9 in thyroid carcinoma. This study showed that the overexpression of HOXA9 significantly enhances *in vitro* normal cell migration and invasion and that tumour cell migration and invasion can be inhibited by downregulating HOXA9. Our results thus showed that HOXA9 could be related to thyroid cell migration an invasion.

As stated, HOXA9 is an upstream regulator of RUNX2. Therefore, we investigated whether its effect on enhanced calcification and carcinogenesis occurs through RUNX2. Thus, we downregulated RUNX2 in two types of thyroid cell lines with or without HOXA9 overexpression. Our results indicated that ALP activity, mineralization behaviour, and cell migration and invasion capacity were all significantly decreased upon RUNX2 downregulation. These results were similar to a previous report showing that the downregulation of RUNX2 can impair migration and invasion in thyroid tumour cells^26^. However, all of these activities were increased with HOXA9 overexpression, as compared to those in the RUNX2-knockdown only groups. These results showed that HOXA9 might enhance the calcification and tumour invasion of PTC not only via RUNX2, but also independently of this marker.

In conclusion, HOXA9, a positive regulator of RUNX2, can enhance calcification and cancer migration and invasion ability in PTC, dependent and independent of RUNX2. Our data improve the understanding of the molecular mechanisms of microcalcification and tumorigenesis in PTC and might lead to the development of novel diagnostic or prognostic biomarkers for this disease.

## Methods

### Cell culture

The human normal thyroid cell line Nthy-Ori 3-1 and PTC cell line BHP10-3 were maintained in complete Roswell Park Memorial Institute 1640 medium (RPMI1640; Welgene, Gyeongsan, Korea) with 10% foetal bovine serum (FBS; Gibco BRL) and 1% antibiotic-antimycotic (Gibco, New York, NY); the other PTC cell line TPC1 was grown in Dulbecco’s Modified Eagle Medium/Nutrient Mixture F-12, 1:1 Mixture (DMEM/F12; Welgene, Gyeongsan, Korea) with 10% FBS and 1% antibiotic-antimycotic. For osteoblast differentiation, cells were plated at 1.0 × 10^4^ cells/well in 48-well plates and used for calcification assays and for PCR; 2.0 × 10^4^ cells were seeded in a 60-mm plate and cells were induced by osteogenic medium after 24 h (for cell adherence; day 0). Medium was changed every 2 days. All cells were incubated in a humidified atmosphere of 5% CO_2_ at 37 °C.

### Candidate regulators of RUNX2

To discover a novel gene that regulates RUNX2, we used the transcription factor search website http://www.cbrc.jp/research/db/TFSEARCH.html with the 3000-bp *RUNX2* promoter and identified putative upstream markers. CTNNBIP1, DLX3, HOXA9, NKX2-5, NKX3-2, RUNX1, and SOX9, with scores greater than 95.00, were selected as candidates.

### Quantitative reverse-transcription PCR (qRT-PCR) and real-time PCR

To determine mRNA expression levels of the bone metabolic markers *RUNX2*, *IBSP*, and *BGLAP* (Osteocalcin) and candidate genes including *CTNNBIP1*, *DLX3*, *HOXA9*, *NKX2-5*, *NKX3-2*, *RUNX1*, and *SOX9*, qRT-PCR and real-time PCR were performed. To normalize the efficiency of real-time RT-PCR reactions, human β-actin was used as a standard. Total RNA was extracted from cultured cells in osteogenesis medium at 0, 3, and 8 days cells using TRIzol reagent (Invitrogen, Carlsbad, USA) following the manufacturer’s instructions and quantified using a spectrophotometer (Beckman Coulter, Brea, USA). Extracted RNA was subsequently reverse transcribed at 42 °C for 1 h using a premix kit with oligo-dT as a primer (iNtRON Biotechnology, Seongnam, Korea). Next, 27–34 cycles of qRT-PCR were performed using a Maxime PCR PreMix kit (i-StarTaq; iNtRON Biotechnology, Seongnam, Korea). All real-time PCR measurements were performed using the ABI Prism 7000 Sequence Detection System (Applied Biosystems; Foster City, USA). All PCR amplifications (25 cycles) were performed in a total volume of 25 μl containing 150 ng cDNA using the SYBR Green I qPCR kit (TaKaRa, Shiga, Japan) according to the manufacturer’s recommendations. The amplification parameters were in accordance with the manufacturer’s recommendations. By normalizing to β-actin, relative quantification of gene expression was performed using the comparative threshold (Ct) method as described by the manufacturer (Applied Biosystems). The values were expressed as fold-change relative to control levels. Relative gene expression was displayed as 2−ΔCt (ΔCt = Ct target gene − Ct β-actin). Fold-change was calculated as 2−ΔΔCt (ΔΔCt = ΔCt control − Ct treatment). PCR primers are described in Table 1.

**Table 1:** Primers for qRT-PCR and real-time PCR.

Gene	Primer (qRT-PCR)
β-actin	5′-CCTAAAAGCCACCCCACTTC-3′
	5′-AGGGAGACCAAAAGCCTTCA-3′
RUNX2	5′-TTACTTACACCCCGCCAGTC-3′
	5′-TATGGAGTGCTGCTGGTCTG-3′
HOXA9	5′-CCACGCTTGACACTCACACT-3′
	5′-CAGTTCCAGGGTCTGGTGTT-3′
**Gene**	**Primer (real-time PCR)**
β-actin	5′-CAAGATCAACCGGGAAAAGA-3′
	5′-CTGAGGCATAGAGGGACAGC-3′
RUNX2	5′-TTACTTACACCCCGCCAGTC-3′
	5′-CACTCTGGCTTTGGGAAGAG-3′
Bsp	5′-AACCTACAACCCCACCACAA-3′
	5′-CGTACTCCCCCTCGTATTCA-3′
BGLAP	5′-GACTGTGACGAGTTGGCTGA-3′
	5′-CTGGAGAGGAGCAGAACTGG-3′
CTNNBIP1	5′-ACCTTTCCCATCATCGTGAG-3′
	5′-AATCCACTGGTGAACCAAGC-3′
DLX3	5′-AGGCCTAGTTCCTCCTGAGC-3′
	5′-CCTCGTCATGATGTCCACTG-3′
HOXA9	5′-CAATAACCCAGCAGCCAACT-3′
	5′-CAGTTCCAGGGTCTGGTGTT-3′
NKX2-5	5′-GTCAAGCCGCTCTTACCAAG-3′
	5′-TTGTCCGCCTCTGTCTTCTC-3′
NKX3-2	5′-GACGCAGGTGAAAATCTGGT-3′
	5′-ACCTTTACGGCCACCTTCTT-3′
Runx1	5′-GGCTGGCAATGATGAAAACT-3′
	5′-CCGACAAACCTGAGGTCATT-3′

### Luciferase reporter and ChIP assays

To analyse *RUNX2* promoter activity, Nthy-Ori 3-1, TPC1, and BHP10-3 cells were plated on 24-well plates at a density of 2 × 10^4^ cells/well 1 day before transfection. Plasmid DNA was mixed with TransIT-2020 (Mirus, Madison, WI) and transfected into the cells following the manufacturer’s protocol. After 48 h, cells were washed twice with 1× PBS and then lysed in reporter lysis buffer (Promega, Madison, WI). Cell extracts were subjected to assays using a luciferase assay system (Promega, Madison, WI) according to the manufacturer’s instructions. Luciferase activity was measured in triplicate, averaged, and then normalized to β-galactosidase activity using o-nitrophenyl-β-D-galactopyranoside (Sigma-Aldrich) as a substrate.

ChIP assays were performed with a ChIP kit (Upstate Biotechnology, Lake Placid. NY), with modified instructions from the manufacturer, using antibodies against HOXA9 (Proteintech, Rosemont, USA) or control IgG (Santa Cruz Biotechnology, Santa Cruz, CA). The precipitated DNA was subjected to PCR amplification with specific primers for the *RUNX2* P1 promoter region, which contains HOXA9-binding sites. The following primers were used for PCR: RUNX2 P1 sense, 5′-GCAAAAAGGCAGAGGTTGAG-3′; RUNX2 P1 antisense, 5′-CCCCCTTGCTCTTTCTCTCT-3′.

### Plasmids, lentivirus packaging, and stable cell lines

We purchased full-length HOXA9wt, pOTB7-HOXA9 from the Korea Human Gene Bank, Medical Genomics Research Center, KRIBB, Korea. *HOXA9* was subcloned into the pcDNA3.1 (+) vector (Invitrogen) by ligating NheI–XhoI fragments to generate the pcDNA-HOXA9 expression construct. pcDNA-HOXA9 was confirmed by DNA sequencing. Stable cell lines were established by transfecting HOXA9 plasmids into thyroid cells and selecting with G418 at a concentration of 400 µg/ml.

Lentivirus harbouring HOXA9 or RUNX2 or vector controls were designed and packaged by Merck (Darmstadt, Germany). Lentiviruses were packaged in HEK-293T cells and collected from the medium supernatant. Stable cell lines were established by infecting thyroid cells with HOXA9- or RUNX2-expressing lentiviruses and selecting with puromycin at a concentration of 10 µg/ml.

### Western blotting

Cell lysates were extracted in 0.1 M NaCl, 0.01 M tris–HCl (pH 7.6), 1 mM EDTA (pH 8.0), 1 mg/ml aprotinin, and 100 mg/ml PMSF; protein concentrations were determined by a Bio-Rad protein assay. Protein (50 μg) was boiled at 95 °C in sodium dodecyl sulphate (SDS) sample buffer for 5 min, electrophoresed on 10% or 12% SDS-PAGE gels, and transferred to polyvinyldifluoridine membranes. Then, blots were incubated overnight at 4 °C with anti-HOXA9 (Abcam, Cambridge, UK, #ab191178; 1:1000), anti-RUNX2 (Abcam, Cambridge, UK, #ab23981; 1:1000), or anti-β-actin (Bethyl, Montgomery, Texas, USA, #A300-491A; 1:8000) antibodies. Membranes incubated with anti-goat or anti-rabbit secondary antibody (Santa Cruz Biotechnology; 1:5000) at room temperature for 60 min. Protein band signals were visualized using an ECF western blotting kit (Amersham Biosciences, Piscataway, NJ, USA) and detected with an LAS3000 luminescent image analyzer (Fuji Photo Film).

### ALP assays and ARS staining

For ALP staining, cultured cells were fixed in 10% formalin for 10 min, permeabilized for 30 min with 0.1% triton-100 in 1× PBS, and treated with nitro blue tetrazolium and 5-bromo-4-chloro-3-indolyl phosphate for 10–30 min. To measure calcium deposition in the extracellular matrix, 200 μl of extraction solution was added and samples were incubated overnight at 4 °C. ALP activity was measured in total cell lysates after homogenization in a buffer containing 1 mM tris-HCl (pH 8.8), 0.5% triton X-100, 10 mM Mg^2+^, and 5 mM p-nitrophenyl phosphate as substrates. The absorbance was read at 405 nm (BioTek, USA).

For ARS staining, cultured cells were fixed in 70% ethyl alcohol for 1 h. After washes with 1× PBS, cells were stained with 40 mM ARS solution (pH 4.2) for 10 min to stain calcium deposits. Cells were then washed five times with distilled water followed by 1× PBS for 15 min and non-specific stained cells were removed. To quantify the degree of mineralization, cells were extracted using 10% (w/v) cetylpyridinium chloride in 10 mM sodium phosphate (pH 7.0). The concentration was evaluated by measuring the absorbance at 562 nm with a multiplate reader using an Alizarin red S standard curve in the same solution. All values are expressed as fold-change relative to the control.

### Wound healing assays

Thyroid cells were seeded in 6-well plates (6 × 10^4^ cells/well) with 2 ml of complete RPMI1640 or DMEM/F-12. At 24 h, the monolayers were mechanically disrupted with a pipette tip to produce a clean uniform scratch. The assay was performed three times and wells were photographed every 12 h to monitor the closing of the wound. The number of migrating cells into the wound was counted under a microscope. Experiments were repeated three times.

### Invasion assays

Matrigel was coated on 24-well chambers (Corning, NY, USA, #3415), and thyroid cells were transferred at a density of 3 × 10^4^ on top of the Matrigel-coated chambers in serum-free media after drying the Matrigel. After 24 h of incubation, non-invading cells were removed by rinsing, and invaded cells were fixed with ice-cold 100% methanol and stained with 0.5% crystal violet. The number of invading cells was manually counted in four randomly chosen fields under a microscope and images were captured at ×100 magnification. Experiments were repeated three times.

### Statistical analysis

The statistical significance of differences was assessed by performing a Student’s t-test. *P* < 0.05 was considered significant. Results are expressed as the mean ± SEM.

## Supplementary information

Supplementary figure

## Data Availability

The datasets used and/or analysed during the current study are available from the corresponding author upon reasonable request.
