# Author Correction: Transcription Factor HOXA9 is Linked to the Calcification and Invasion of Papillary Thyroid Carcinoma

**DOI:** 10.1038/s41598-020-59302-x

**Published:** 2020-02-06

**Authors:** Yilan Jin, Hyeung Kyoo Kim, Jeonghun Lee, Euy Young Soh, Jang-Hee Kim, Insun Song, Yoon-Sok Chung, Yong Jun Choi

**Affiliations:** 1 Department of Endocrinology and Metabolism, Ajou University School of Medicine, Suwon, South Korea; 2 Department of Surgery, Ajou University School of Medicine, Suwon, South Korea; 3 Department of Pathology, Ajou University School of Medicine, Suwon, South Korea; 4 School of Biological Sciences, Seoul National University, Seoul, South Korea

Correction to: *Scientific Reports* 10.1038/s41598-019-43207-5, published online 01 May 2019

This Article contains errors.

In Figure 2a, the incorrect images were used for HOXA9-knockdown Nthy-Ori 3-1 cells, at 10 days, and BHP10-3 control cells, at 7 days.

In Figure 3a, the incorrect images were used for Nthy-Ori 3-1 control cells, and HOXA9-knockdown Nthy-Ori 3-1 cells, at 0 hours.

In Figure 4b, the incorrect image was shown for over-expressing HOXA9 and RUNX2-knockdown BHP10-3 cells, at 7 days.

The correct Figures 2, 3 and 4 appear below as Figs. 1–3 respectively.

**Figure 1:**
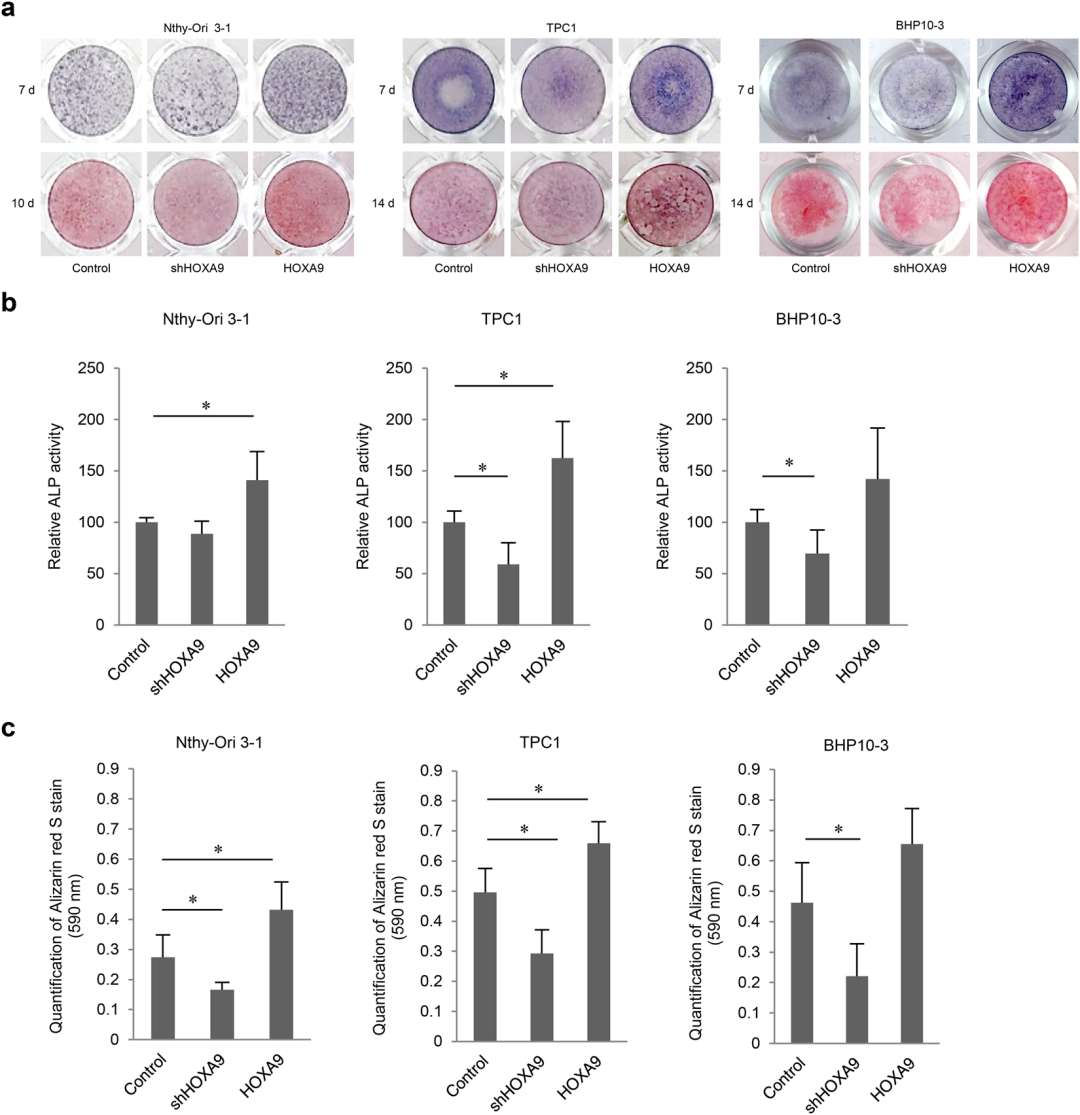
.

**Figure 2:**
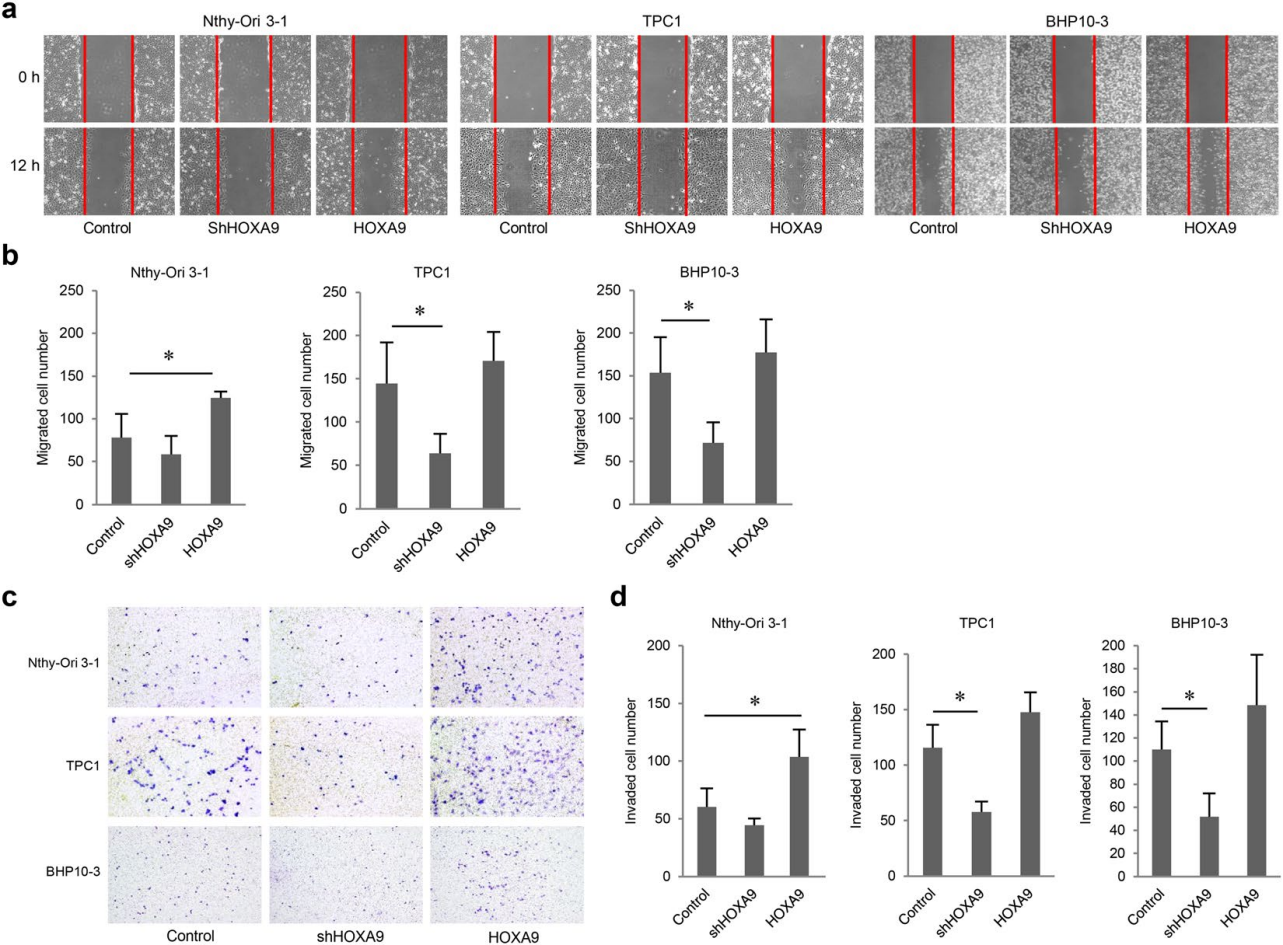
.

**Figure 3:**
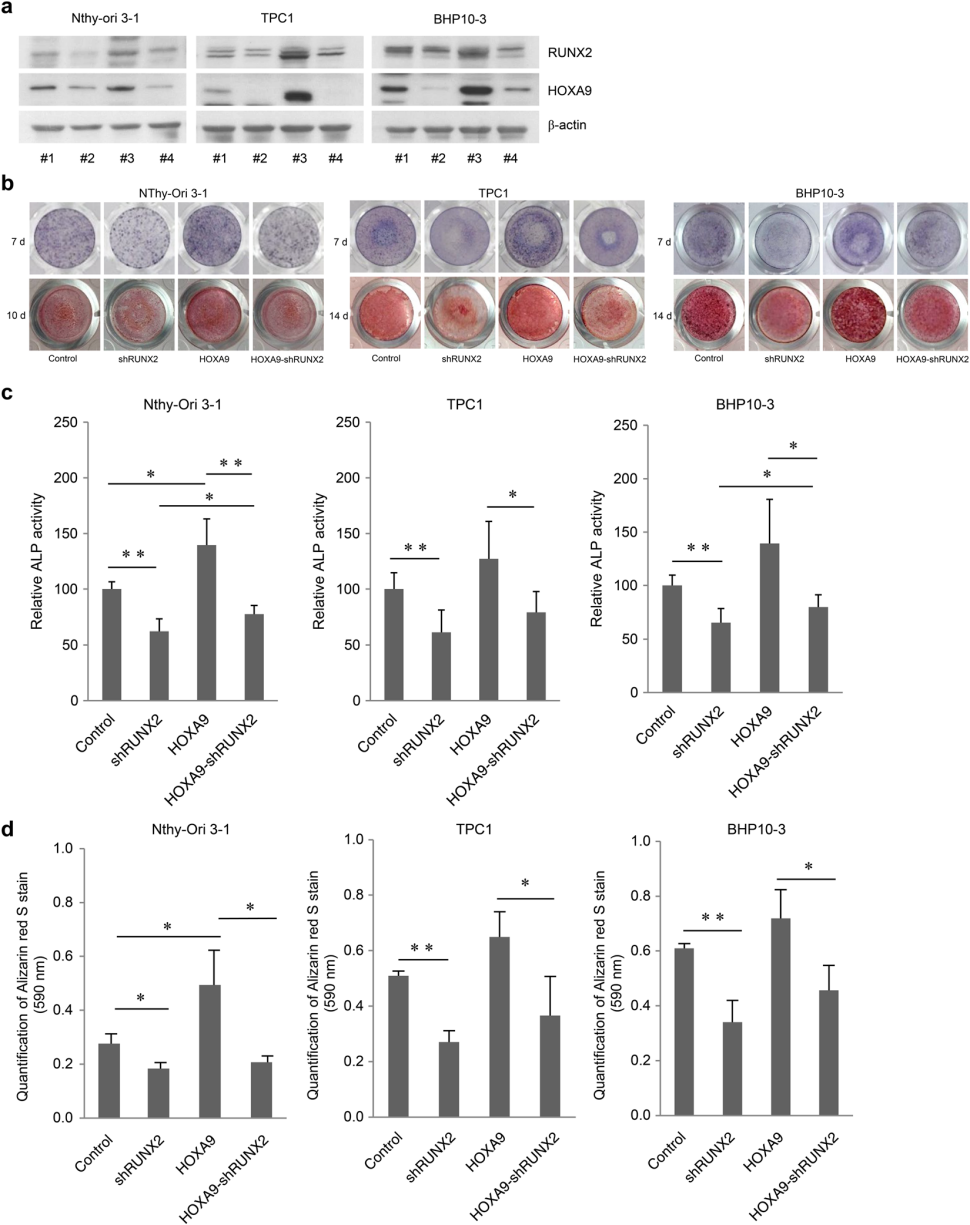
.

The main conclusions of the Article are unaffected by these changes.

