# Supplementary material for: Transcription Factor HOXA9 is Linked to the Calcification and Invasion of Papillary Thyroid Carcinoma
Source: Sci Rep. 2019 May 1;9:6773. doi: 10.1038/s41598-019-43207-5 (PMC6494860; doi:10.1038/s41598-019-43207-5)
Supplement: Supplementary file 1 — Supplementary figure [file 41598_2019_43207_MOESM1_ESM.pdf]

Transcription Factor HOXA9 is Linked to the Calcification and Invasion of Papillary Thyroid Carcinoma

Yilan Jin[1], Hyeung Kyoo Kim[2], Jeonghun Lee[2], Euy Young Soh[2], Jang-Hee Kim[3], Insun Song[1,4], Yoon-Sok Chung[1], Yong Jun Choi[1,*]

[1]Department of Endocrinology and Metabolism, Ajou University School of Medicine, Suwon, South Korea

[2]Department of Surgery, Ajou University School of Medicine, Suwon, South Korea

[3]Department of Pathology, Ajou University School of Medicine, Suwon, South Korea

[4] School of Biological Sciences, Seoul National University, Seoul, South Korea

*Address for correspondence to

Yong Jun Choi, MD, PhD

Assistant Professor

Department of Endocrinology and Metabolism, Ajou University School of Medicine

164 Worldcup-ro, Yeongtong-gu, Suwon, 16499, Korea

Tel.: +82-31-219-4491

Fax: +82-31-219-4497

E-mail: colsmile@hanmail.net

Supplementary Figure S1

Full-size original blots of cropped blots shown in Figures

Fig. 1f

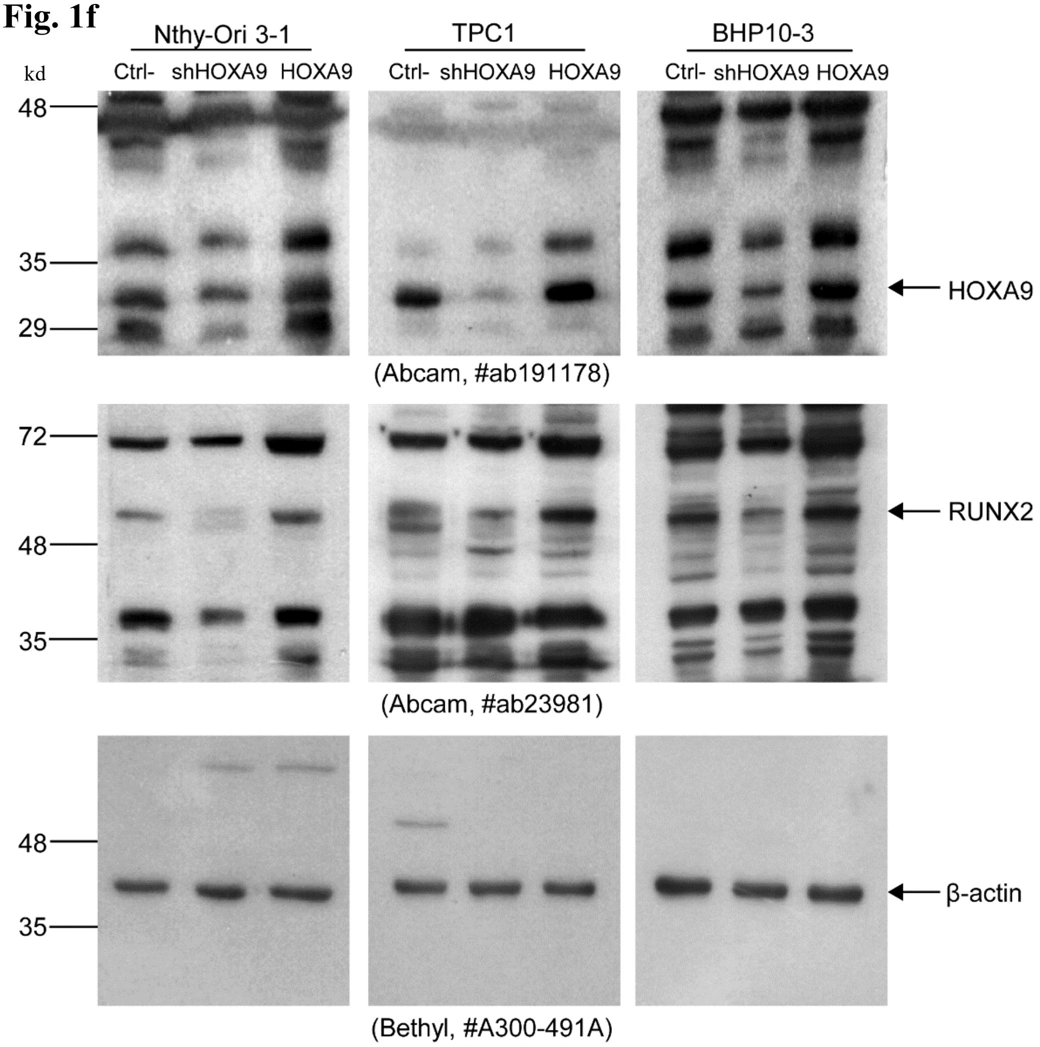

Full-length blots for cropped blots in Fig. 1f.

Supplementary Figure S2

Full-size original blots of cropped blots shown in Figures

Fig. 5a

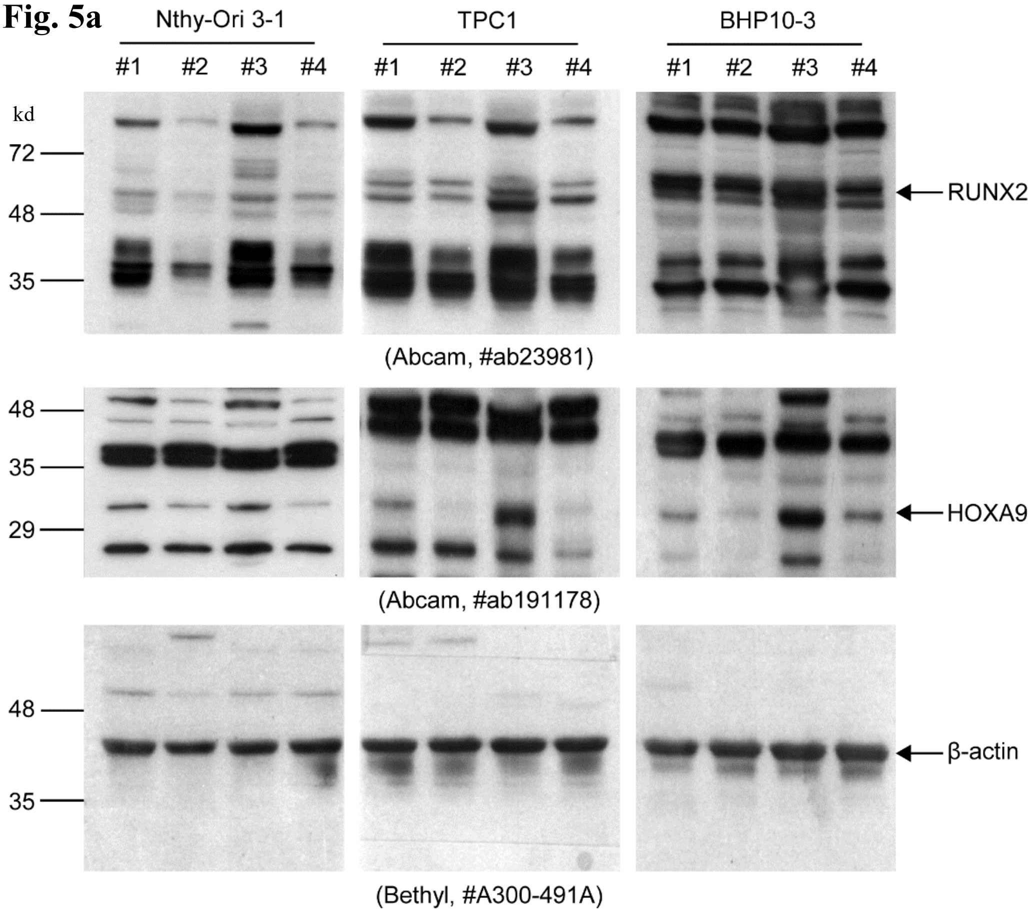

Full-length blots for cropped blots in Fig. 5a.
